# Hemodynamics and Hemorrhagic Transformation After Endovascular Therapy for Ischemic Stroke

**DOI:** 10.3389/fneur.2020.00728

**Published:** 2020-07-17

**Authors:** Andrew Silverman, Sreeja Kodali, Kevin N. Sheth, Nils H. Petersen

**Affiliations:** Department of Neurology, Yale School of Medicine, New Haven, CT, United States

**Keywords:** thrombectomy, blood pressure, stroke, autoregulation dysfunction, neurocritical care management

## Abstract

Hemorrhagic transformation remains a potentially catastrophic complication of reperfusion therapies for the treatment of large-vessel occlusion ischemic stroke. Observational studies have found an increased risk of hemorrhagic transformation in patients with elevated blood pressure as well as a high degree of blood pressure variability, suggesting a link between hemodynamics and hemorrhagic transformation. Current society-endorsed guidelines recommend maintaining blood pressure below a fixed threshold of 180/105 mmHg regardless of thrombolytic or endovascular intervention. However, given the high recanalization rates with mechanical thrombectomy, it is unclear if the same hemodynamic goals from the pre-thrombectomy era apply. Also, individual patient factors such as the degree of reperfusion, infarct size, and collateral status likely need to be considered. In this review, we will discuss current evidence linking hemodynamics to hemorrhagic transformation after mechanical thrombectomy. In addition, we will review the clinical relevance of cerebral autoregulation in stroke, highlighting recent studies that have harnessed autoregulatory physiology to define and trend individualized limits of autoregulation. This review will go on to emphasize the translatability of this approach to stroke management. Finally, we will discuss novel statistical approaches like trajectory analysis to post-thrombectomy hemodynamics.

## Introduction

Hemorrhagic transformation (HT) is a feared complication of acute ischemic stroke and is independently associated with neurological deterioration and worse functional outcomes (1–4). Accurate prediction and triage of patients at risk for HT would be of tremendous value, and yet the underlying mechanisms and potential biomarkers of HT remain elusive. While animal and human studies have invoked pathomechanisms involving neuroinflammation, neurovascular unit impairment, blood brain barrier disruption, and vascular remodeling, this clinically oriented review will focus on cerebral autoregulation and optimal blood pressure (BP) management following endovascular thrombectomy (EVT) for large-vessel occlusion (LVO) acute ischemic stroke (5, 6).

Mechanical thrombectomy preceded by intravenous thrombolytics has become standard of care treatment in stroke patients with acute ischemia secondary to LVO (7). This shift occurred after 2015, a year that witnessed five randomized trials (MR CLEAN, ESCAPE, SWIFT PRIME, REVASCAT, and EXTEND IA), showing the efficacy of EVT over standard medical care (8–12). A subsequent meta-analysis (HERMES) included a total of 1,287 patients and demonstrated a significant reduction in 90-days disability compared to controls, though 90-days mortality did not differ between the two study populations (7). Two additional trials (DAWN, DEFUSE-3) were published in 2018. They provided evidence that thrombectomy can be offered up to 24 h after symptom onset in selected patients with a mismatch between infarct size and clinical deficit (13, 14).

In all seven of these major trials, the rates of symptomatic HT were key safety outcomes, reported as serious adverse events following treatment. In the first five studies that looked at EVT in the early window (up to 12 h), symptomatic HT in the treatment group ranged from 0 to 7.7%. Of note, in these five studies, most patients (>80%) in both intervention and control groups received intravenous thrombolysis in addition to EVT. In both extended time window trials, symptomatic hemorrhagic complications occurred in 6–7% of patients in the treatment group. The DEFUSE 3 trials' rates of symptomatic intracranial bleeding did not differ between the EVT and control group (7 vs. 4%, respectively; *P* = 0.75) (13). Five patients with symptomatic HT in the EVT group died, compared with two in the control group. In the DAWN trial, the rates of symptomatic intracranial bleeding did not significantly differ between the EVT and control groups (6 vs. 3%, respectively; *P* = 0.50) (14). The HERMES pooled analysis of patient-level data concluded that the rates of symptomatic intracranial hemorrhage are not higher in patients receiving EVT than in patients receiving medical therapy alone (4.4 vs. 4.3%, respectively; risk difference 0.1%), suggesting that reperfusion alone may not be the primary driver of symptomatic HT (7). Observational studies have shown an increased risk of HT with sustained post-procedural hypertension and higher BP variability (15). Interestingly, mean systolic BP (SBP) was lower among patients with successful reperfusion, indicating a possible difference in the threshold for reperfusion injury depending on recanalization status. Furthermore, radiographic hemorrhagic infarction (HI) is common following EVT and has been associated with poor outcome, thereby questioning the purported benign nature of HI (4). While these studies suggest a possible role of hemodynamics in the development of HT, they do not prove a causal relationship. Identification of patients at risk for HT (both radiographic and symptomatic) may allow for early preventative strategies like BP control post-EVT.

## Blood Pressure Management Following Thrombectomy

Current American Heart Association guidelines recommend maintaining BP < 180/105 mmHg for all patients treated with intravenous thrombolysis or EVT to promote perfusion to ischemic territories while mitigating potential risks of intracranial hemorrhage. Still, guidelines acknowledge a lack of prospective trials to substantiate this position, and the language of these consensus statements reflects this uncertain area of care: “In patients who undergo mechanical thrombectomy, it is reasonable to maintain the BP ≤ 180/105 mmHg during the first 24 h after the procedure. In patients who undergo mechanical thrombectomy with successful reperfusion, it might be reasonable to maintain BP at a level <180/105 mmHg.” (16). Randomized controlled trials are unavailable, and the evidence in support of these recommendations is moderate to weak (class of recommendation IIa&IIb, level of evidence B-NR). Furthermore, trial protocols regarding post-procedural BP control in the studies that contributed to guideline development were vague, and BP management likely varied across sites. The vast majority of patients enrolled in under 6-h randomized trials received intravenous thrombolytic therapy, and the trial protocols stipulated management according to local guidelines with pressures generally under 180/105 mmHg for the first 24 h after the procedure. Only two trial protocols provided additional recommendations. The ESCAPE protocol states that systolic BP ≥ 150 mmHg is probably useful in promoting and sustaining adequate collateral flow while the artery remains occluded (9). The protocol further states that controlling pressure once reperfusion has been achieved, aiming for normal pressures, is a reasonable route for individual patients. Second, the DAWN protocol endorses systolic pressures under 140 mmHg in the first 24 h for subjects who achieve successful reperfusion (17). As a result of the limited data, current management strategies are based on guidelines that favor a one-size-fits-all approach that neglects the heterogeneity of stroke and differences in individual patient characteristics. The care of patients with stroke is, therefore, poorly individualized.

Despite the efficacy of EVT, many patients with LVO stroke still suffer morbidity, mortality, and functional dependence in longitudinal studies (7, 18). Observational studies, including a recent meta-analysis, have shown higher rates of HT, worse outcomes, and increased mortality in patients with higher peak SBP values or hemodynamic variability in the first 24 hours after EVT (15, 19–21). However, it remains unclear if post-procedural hypertension is simply an epiphenomenon, or if it reflects a valid therapeutic target. In a recent multicenter study of 1,245 patients who achieved successful reperfusion after EVT, Anadani et al. divided patients into three groups based on SBP goal in the first 24 h post-EVT. The investigators found that higher SBP targets were associated with higher odds of symptomatic intracranial hemorrhage, mortality, and hemicraniectomy (22). The results agree with earlier findings by Goyal et al., who published a single-center experience after the implementation of more aggressive BP control following successful EVT. Compared to patients treated with permissive hypertension (<180 mmHg), those treated with moderate (<160 mmHg) and intensive (<140 mmHg) BP control showed improved functional outcome and lower mortality at three months (19). Although we currently lack rigorous clinical evidence, these studies, as well as compelling conceptual reasons, suggest that BP optimization may represents a post-EVT neuroprotective strategy.

Indeed, while a higher BP may be beneficial in patients with incomplete reperfusion by promoting perfusion to ischemic territories and the penumbra, it could lead to relative hyperperfusion. Such hyperperfusion could cause cerebral edema and hemorrhage in those patients with complete reperfusion. This phenomenon is well-described in chronic ischemia after carotid revascularization (via endarterectomy or stenting) but may also occur in acute stroke (23–25). For example, Hashimoto *et al*. reported cerebral hyperperfusion syndrome in a 77-year-old patient with acute internal carotid and middle cerebral artery occlusions. Due to the patient's neurologic deterioration, the authors suggest that it is essential to routinely monitor regional oxygen saturation with near-infrared spectroscopy, evaluate cerebral blood flow, and maintain antihypertensive therapy to prevent hyperperfusion after revascularization (25). It is also possible that this complication is more prevalent than the handful of published case reports might suggest. Following recanalization, lower BP targets may be warranted to decrease reperfusion injury and promote penumbral recovery. Nevertheless, optimal, personalized BP targets remain undefined. To complicate the matter, individual patient factors such as degree of reperfusion, infarct size, concomitant carotid revascularization, antithrombotic therapy, and hemodynamic status likely need to be considered. Because of these factors, there is a high degree of practice variation in BP management following EVT (26).

Recent studies have shown that real-time autoregulation monitoring can be used to identify a dynamic BP range in individual patients at which autoregulation is optimally functioning (27–31). Such an autoregulation-derived, personalized BP range may provide a favorable physiologic landscape for the acutely injured brain. Accordingly, the following section will review the use of cerebral autoregulation monitoring in patients with acute ischemic stroke, highlighting the hypothesis that exceeding a personalized upper limit of autoregulation predisposes patients to reperfusion injury and HT (27, 29)

## Cerebral Autoregulation and Blood Pressure Personalization

Cerebral autoregulation describes the intrinsic capacity of the cerebral vasculature to preserve stable blood flow in the face of systemic BP changes (or, more precisely, cerebral perfusion pressure changes) (32). Autoregulatory capacity in acute stroke is critical for the maintenance of stable blood flow to the ischemic penumbra and avoidance of excessive hyperperfusion (33, 34). There is fairly widespread agreement that stroke is associated with impaired autoregulation, even in cases of minor stroke (33–35). This impairment may exist ipsilateral to the stroke site in a focal fashion, or globally throughout both hemispheres (34). Interestingly, Immink et al. reported dynamic autoregulatory disturbance ipsilateral to middle cerebral artery (MCA) territory strokes but bilaterally in lacunar ischemic strokes (36). These results were bolstered in more recent analyses by Guo et al., showing that dynamic autoregulatory markers were impaired ipsilaterally in a stroke of large artery atherosclerosis but bilaterally in stroke of small artery occlusion (37). Petersen et al. then examined autoregulation on a more longitudinal basis, reporting dynamic autoregulatory failure up to 1 week following acute LVO strokes in the MCA. More specifically, this investigation showed that the autoregulatory parameter phase was lower in the affected cerebral hemisphere compared to the contralateral hemisphere, indicating an impaired ability to buffer against BP fluctuations (38).

Furthermore, in stroke patients with impaired autoregulation, recovery tends to be delayed for up to 3 months, underlining the clinical relevance of autoregulation in stroke research (35, 39). That said, only a handful of studies have looked at functional outcome prognostication with respect to autoregulation physiology in stroke. For example, Reinhard et al. enrolled 45 patients within 48 h of LVO MCA strokes and showed that ipsilateral lower phase shifts were related to worse functional outcomes (40). In light of the prolonged enrollment timeframe, the authors conceded that autoregulatory impairment might reflect initial stroke severity, rather than functioning as an independent contributing factor to outcome. To help resolve this question, Castro et al. measured autoregulation in 30 patients with LVO MCA ischemic stroke within 6 h of symptom onset (39). This report demonstrated that autoregulatory impairment operated as a statistically independent predictor of functional autonomy at the 90-days endpoint (odds ratio 14.0, 95% confidence interval 1.7–74.0; *P* = 0.013). In yet another study, these authors reported that final infarct volume is significantly lower in patients with preserved autoregulation in a similar acute window post-stroke (41). In a review summarizing these findings, Castro *et al*. conclude that early autoregulatory measures wield considerable import in the guidance of acute stroke management, secondary injury prevention, and outcome improvement (35).

Autoregulatory physiology has thus been invoked as a biological avenue with possible deterrent and restorative benefits concerning HT and associated neurologic worsening. In an invasive neuromonitoring study, Dohmen et al. enrolled 15 patients with MCA ischemic strokes and calculated the cerebral perfusion pressure-oxygen reactivity index (COR) (42). They found COR indices were higher (worse) in the eight patients with malignant courses (i.e., massive brain edema) compared to the seven patients with relatively benign courses. The study concludes that dysautoregulation appears to play an essential role in the development of cerebral edema. In a study mentioned above, Castro et al. calculated cerebrovascular resistance, coherence, gain, and phase in 46 patients within 24 h of MCA ischemic stroke (41). At admission, phase was lower (indicative of worse autoregulation) in patients with HT. Also, progression to edema was related to lower cerebrovascular resistance values and increased blood flow velocities at the initial presentation. These lower resistances, the authors submit, reflect paradoxical cerebral vasodilation, as cerebrovascular resistance is equal to the quotient between mean arterial pressure and mean flow velocity (CVR = MAP/MFV). Thus, they argue that breakthrough hyperperfusion and microvascular injury may underlie the development of malignant edema and HT.

**Figure 1:**
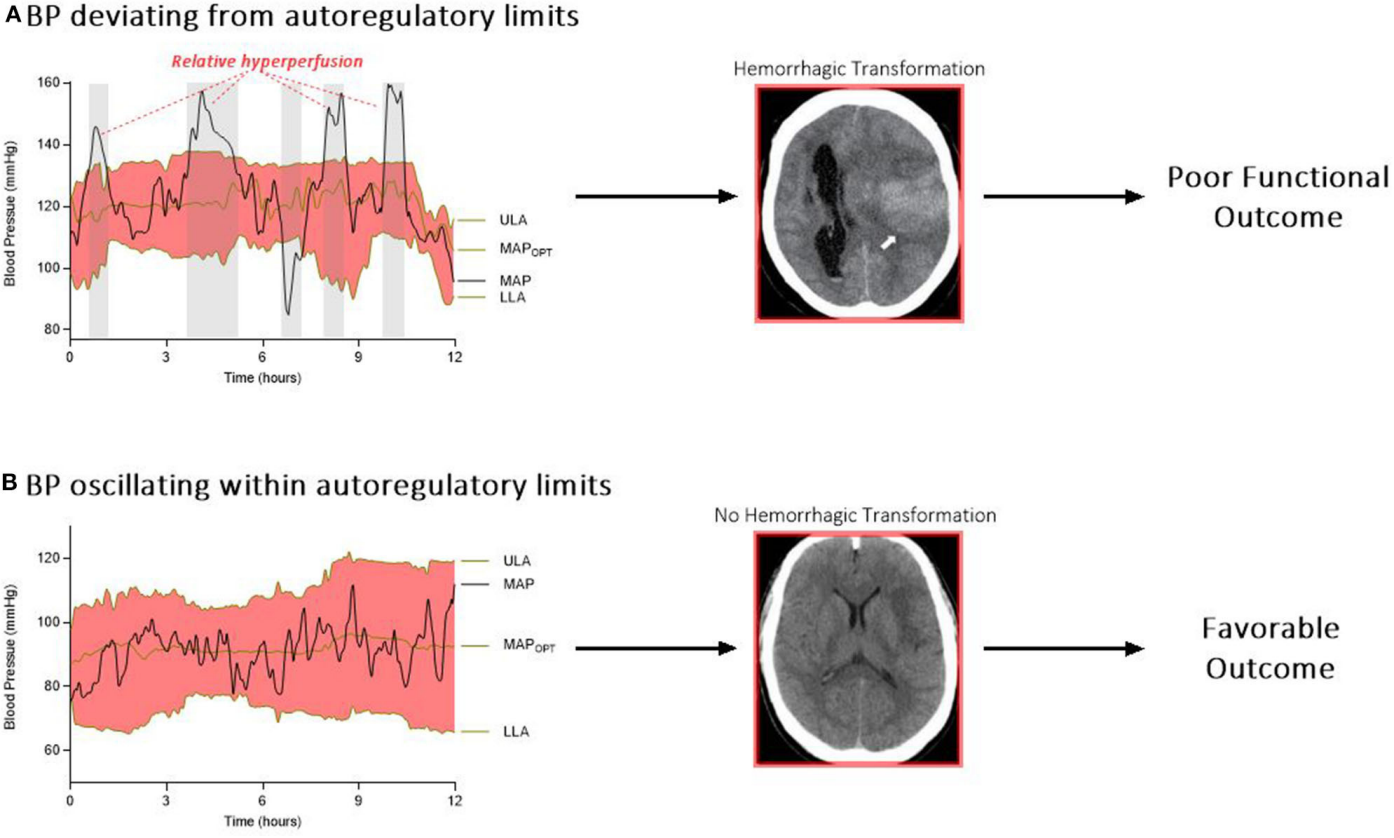
**(A)** Relative hyperperfusion above the upper limit of autoregulation may predispose patients to hemorrhagic transformation and worse outcomes. **(B)** In contrast, patients who oscillate within their personalized limits of autoregulation may be protected from secondary brain injury after stroke. ULA, upper limit of autoregulation; MAP_OPT_, optimum mean arterial pressure; MAP, mean arterial pressure; LLA, lower limit of autoregulation.

Cumulatively, there is substantial evidence for impaired autoregulation after stroke. It follows that an autoregulation-guided approach can be applied to the cerebrovascular hemodynamics of stroke pathophysiology. The Cambridge group has been refining this work over several decades, particularly in patients with traumatic brain injury (43). With this hypothesis in mind, a recent study harnessed autoregulation monitoring to identify and track personalized BP limits in 90 patients undergoing EVT for LVO ischemic stroke (27, 29). This cohort revealed that continuous estimations of optimal BP and autoregulatory limits are feasible in post-EVT care. The study further demonstrated that exceeding individualized autoregulatory thresholds was associated with HT and worse outcome (Figure 1). In more detail, every 10% increase in time spent above the upper limit of autoregulation was associated with a doubling in the odds of shifting toward a more unfavorable 3-months outcome. The study also observed a progressive increase in percent time above this upper limit with worsening grades of HT (11.4% of the time for no HT, 13.5% for hemorrhagic infarctions 1 and 2, and 20.9% for parenchymal hematoma 1 and 2; *P* = 0.03). Also, patients who developed symptomatic intracranial hemorrhage spent more time above the upper autoregulatory limit when compared to patients without this complication (11.9 vs. 24.6%; *P* = 0.1) (29).

This relationship between deviation from the upper autoregulatory limit and outcome is supported by the construct that above the upper autoregulatory limit, the cerebral vasculature functions as a pressure-passive system, in which increases in cerebral blood flow are not counteracted by vasoconstriction (44). This system permits periods of hyperperfusion in the setting of an elevated systemic BP (33). Furthermore, higher cerebral blood flow after reperfusion therapy (measured via arterial spin labeling magnetic resonance imaging) has been shown to increase the risk of HT (45). Several retrospective studies reported an association between sustained hypertension after EVT and HT (15, 46), although others did not unearth this relationship (19, 47). Divergence of autoregulatory capacity among different patients may be at least one explanation for these discordant results.

An additional aim of this post-EVT monitoring study was to compare personalized, autoregulation-guided BP targets with two commonly used clinical approaches: 1) maintaining BP below a fixed, pre-determined value as recommended by current guidelines and 2) stratifying BP thresholds based on reperfusion status (29). Ultimately, there was no association between time spent above any of the fixed SBP thresholds and HT or functional outcome, even after stratifying by reperfusion status. This supplementary analysis was particularly important because optimal BP ranges after EVT are likely influenced by numerous factors; stratifying by reperfusion status alone might not be sufficient. For instance, chronic hypertension and flow-limiting extracranial carotid disease may shift a person's autoregulatory curve toward higher pressures. Aggressively lowering BP after successful EVT in this scenario may result in cerebral hypoperfusion and infarct expansion (48, 49). In comparison, optimal BP ranges could shift toward lower pressures in patients without hypertension or pre-existing large-vessel disease. Overall, then, these results argue for future research in prospective, multicenter, and randomized trials. Finally, another interesting avenue of investigation revolves around the question of restoring dysautoregulation by dynamically adjusting BP. In other words, by targeting an optimum BP within autoregulatory limits, intensivists may be able to shift patients to a more favorable position on the autoregulatory curve, but this hypothesis remains untested.

## Blood Pressure Trajectory Analysis After Stroke

In addition to autoregulation monitoring, researchers in recent years have applied innovative statistical tools to study BP data in the acute window post-stroke. For instance, in 2018, Kim et al. used trajectory modeling to examine longitudinal BP data from a prospective multicenter registry of 8,376 stroke patients (50). Their characterization of post-stroke BP courses has been hitherto a missing element in the field. In their work, the authors applied the TRAJ procedure from SAS software to separate heterogeneous, longitudinal BP data into trajectory groups with similar patterns. This analysis identified the optimal number and shape of trajectories; it then assigned patients to estimated trajectory groups. Five distinct BP trajectories were generated over the acute period following stroke. The risk of recurrent stroke, myocardial infarction, or death was greater in patients who fell into the acutely elevated or persistently high BP trajectory groups.

In 2019, Li et al. published a *post-hoc* BP trajectory analysis of a large BP lowering trial in 4,036 patients with stroke (51). Using similar statistical methods, the authors generated five BP trajectories over seven days following stroke. Patients who sustained high BP over time had significantly higher mortality rates at 3-months and 2-years follow-up. Patients in the experimental arm of the original trial who received BP lowering interventions were more likely found in lower BP trajectories than patients in the control arm, demonstrating that pharmacological intervention can affect a patient's BP trajectory and potentially their outcome. These two studies, then, reaffirm the association between elevated post-stroke BP and poor outcome.

**Figure 2:**
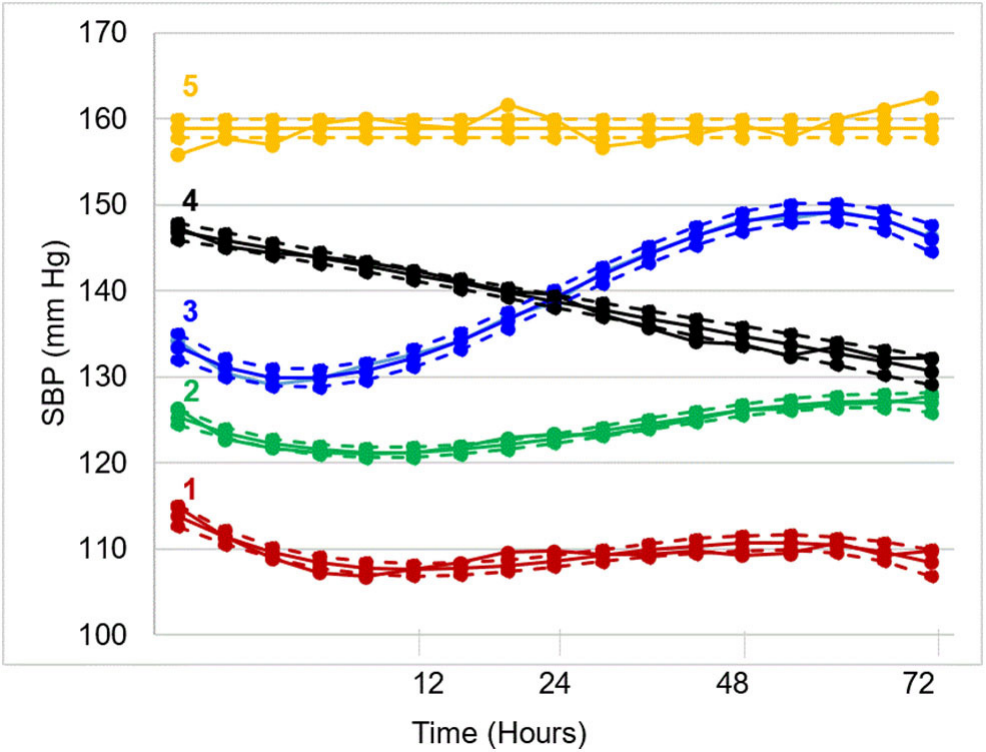
Systolic blood pressure trajectories over 72 h post-EVT. Five distinct trajectories emerged: (1) low (17%), (2) moderate (38%), (3) moderate-to-high (21%), (4) high-to-moderate (17%), and (5) high (7%).

In recent work by Petersen et al., trajectory analysis was conducted on a prospective, multicenter, international cohort of 1,060 patients who underwent EVT for LVO ischemic stroke (52). Five unique post-EVT systolic BP trajectories were generated over 72 h (Figure 2). Compared to patients in the moderate trajectory (2), patients in the acutely elevated (4) and persistently high (5) trajectories had a significantly increased risk of unfavorable functional outcome after adjustment for several covariates (odds ratio 1.6 and 2.5, respectively). While the elevated BP in high trajectory groups may reflect an acute, post-stroke hypertensive response, it may also reflect underlying, untreated hypertension. Patients in higher trajectories had higher rates of hypertension and received more antihypertensive medication pre-admission. Additionally, elevated BP may reflect reperfusion status, as non-recanalized patients were more likely to be in higher trajectory groups. Overall, patients who maintained lower BP trajectories had better 90-days functional outcomes, but this trend was not observed for symptomatic HT. Patients in the acutely elevated (4) trajectory had the highest rate of symptomatic HT, even more than patients in the persistently high trajectory (5). In contrast, patients in the moderate-to-high (3) trajectory (who had the highest rates of in-hospital antihypertensive treatment) had markedly lower rates of symptomatic HT than any other trajectory group. These findings raise questions about alternative mechanisms, such as cerebral edema, through which elevated BP may impact functional outcome.

It is unknown whether lowering a patient's trajectory from persistently high (5) to acutely elevated (4) will improve outcomes, as this retrospective analysis of a prospective cohort was purely observational. However, these findings may help identify ideal candidates for future trials. This work, along with the previously described studies on autoregulation-based BP goals, are hypothesis-generating and aim to identify a subset of patients who may benefit most from post-stroke BP intervention. Additionally, this body of work demonstrates the impact of emerging analytical techniques on understanding post-stroke hemodynamics, prevention of secondary injuries like HT, and more personalized BP management.

## Conclusion

In the era of endovascular thrombectomy, hemorrhagic transformation remains a potentially devastating complication of acute ischemic stroke. Intracranial bleeds after thrombectomy likely occur as a result of a multifactorial process. Still, this clinical review of BP optimization shows that hemodynamic management represents a titratable, neuroprotective avenue in the care of critically ill patients. Exceeding the upper limit of autoregulation may predispose patients to reperfusion injury; maintaining BP within autoregulatory limits may achieve favorable outcomes while avoiding hemorrhagic complications. Additionally, trajectory analysis has the potential to provide more tailored hemodynamic management in the post-thrombectomy intensive care setting.

## Author Contributions

AS and SK contributed in equal parts to the manuscript's concept and design. KS and NP provided critical feedback and revisions for intellectual content.

## Conflict of Interest

The authors declare that the research was conducted in the absence of any commercial or financial relationships that could be construed as a potential conflict of interest.
